# CDDO-Me reveals USP7 as a novel target in ovarian cancer cells

**DOI:** 10.18632/oncotarget.12801

**Published:** 2016-10-21

**Authors:** Dongjun Qin, Weiwei Wang, Hu Lei, Hao Luo, Haiyan Cai, Caixia Tang, Yunzhao Wu, Yingying Wang, Jin Jin, Weilie Xiao, Tongdan Wang, Chunmin Ma, Hanzhang Xu, Jinfu Zhang, Fenghou Gao, Ying-Li Wu

**Affiliations:** ^1^ Hongqiao International Institute of Medicine, Shanghai Tongren Hospital/Faculty of Basic Medicine, Chemical Biology Division of Shanghai Universities E-Institutes, Key Laboratory of Cell Differentiation and Apoptosis of the Chinese Ministry of Education, Shanghai Jiao Tong University School of Medicine, Shanghai, China; ^2^ Institute of Oncology, Shanghai 9th People's Hospital, Shanghai Jiao Tong University School of Medicine, Shanghai, China; ^3^ State Key Laboratory of Phytochemistry and Plant Resources in West China, Kunming Institute of Botany, Chinese Academy of Sciences, Yunnan, China

**Keywords:** CDDO-Me, ovarian cancer, USP7, CETSA, deubiquitinating enzymes

## Abstract

Deubiquitinating enzyme USP7 has been involved in the pathogenesis and progression of several cancers. Targeting USP7 is becoming an attractive strategy for cancer therapy. In this study, we identified synthetic triterpenoid C-28 methyl ester of 2-cyano-3, 12-dioxoolen-1, 9-dien-28-oic acid (CDDO-Me) as a novel inhibitor of USP7 but not of other cysteine proteases such as cathepsin B and cathepsin D. CDDO-Me inhibits USP7 activity via a mechanism that is independent of the presence of α, β-unsaturated ketones. Molecular docking studies showed that CDDO-Me fits well in the ubiquitin carboxyl terminus-binding pocket on USP7. Given that CDDO-Me is known to be effective against ovarian cancer cells, we speculated that CDDO-Me may target USP7 in ovarian cancer cells. We demonstrated that ovarian cancer cells have higher USP7 expression than their normal counterparts. Knockdown of USP7 inhibits the proliferation of ovarian cancer cells both *in vitro* and *in vivo*. Using the cellular thermal shift assay and the drug affinity responsive target stability assay, we further demonstrated that CDDO-Me directly binds to USP7 in cells, which leads to the decrease of its substrates such as MDM2, MDMX and UHRF1. CDDO-Me suppresses ovarian cancer tumor growth in an xenograft model. In conclusion, we demonstrate that USP7 is a novel target of ovarian cancer cells; targeting USP7 may contribute to the anti-cancer effect of CDDO-Me. The development of novel USP7 selective compounds based on the CDDO-Me-scaffold warrants further investigation.

## INTRODUCTION

Ovarian cancer is one of the most common gynecological malignancies in women [1]. Despite great advances in chemotherapy and surgical treatment of this disease, the 5-year survival remains approximately 36% [2]. Over 80% of patients with advanced ovarian cancer will relapse and eventually die of their disease [3]. Thus, identification of novel target to conquer this disease is urgently required.

Protein ubiquitination is a dynamic reversible process. Removal of ubiquitin (Ub) is performed by deubiquitinating enzymes (DUBs), which are grouped into five structurally different DUB enzyme families. Ubiquitin-specific enzymes (USP) constitute the largest subfamily of DUBs, with more than 60 human member proteins. USPs can release poly-Ub chains from proteins targeted for degradation, recycle monomeric Ub, liberate Ub from Ub-fusion precursors, reverse regulatory ubiquitylation and edit inappropriately ubiquitylated proteins [4–5]. USPs, such as USP1 [6], USP2 [7], USP7 [8], USP9x [9] and USP14 [10], were recently shown to contribute to the development of cancer and are emerging as novel cancer drug targets [11–12]. USP7, also known as herpes-associated USP, has been found to be critical in cancer progression because of its influence on the stability of the tumor suppressor p53 [13]. USP7 has a higher binding affinity for MDM2, thereby causing MDM2 stabilization via the antagonisation of its autoubiquitination and the consequent induction of p53 degradation. Therefore, the inhibition of USP7 is predicted to destabilise MDM2 and stabilize p53. Additional substrates of USP7 have been reported; these substrates include claspin [14], FOXO4 [15], PTEN [16], UHRF1 [17]. USP7 exerts both p53-dependent and p53-independent effects on controlling cell proliferation and apoptosis, thereby making USP7 as an attractive target for cancer therapy [8]. However, the possible role of USP7 in ovarian cancer is not yet clear.

Triterpenoids are biosynthesized in plants by the cyclization of squalene. They are used for medicinal purposes in several Asian countries. In addition, ursolic and oleanolic acids have anti-inflammatory, hepatoprotective and anti-tumor activities [18]. The C-28 methyl ester of 2-cyano-3,12-dioxoolen-1,9-dien-28-oic acid (CDDO-Me) is a novel synthetic oleanane triterpenoid. CDDO-Me is currently in the late stages of clinical development for the treatment of chronic kidney disease (diabetic nephropathy) [19–21]. CDDO-Me exhibits cytotoxicity against various cancer cells, including ovarian cancer [22–23]. However, the underlying mechanism of anti-ovarian cancer activity of CDDO-Me is not well understood.

In this study, we demonstrate for the first time that CDDO-Me inhibits USP7 activity by directly interacting with the protein. Furthermore, targeting USP7 contributed to CDDO-Me-induced ovarian cancer cell death, suggesting that USP7 is a novel drug target in ovarian cancer cells.

## RESULTS

### CDDO-Me inhibits USP7 activity *in vitro*

**Figure 1:**
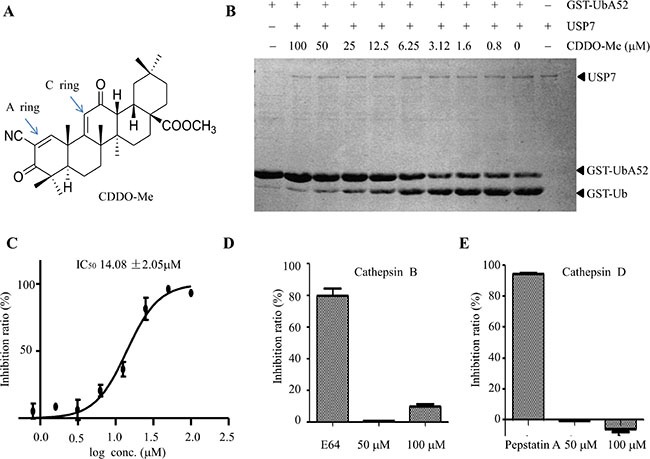
CDDO-Me inhibits USP7 activity *in vitro* (**A**) The chemical structure of CDDO-Me. (**B**) In an *in vitro* gel-based USP7 activity assay, various concentrations of CDDO-Me were pre-incubated with 80 nM USP7 before GST-UBA52 was added. After incubation, the reactions were stopped, and the products were separated by 12% SDS-PAGE and visualized by Coomassie brilliant blue (G250), and the IC_50_ is 14.08 μM (**C**). (**D**–**E**) The effect of 50 and 100 μM CDDO-Me on the activity of cathepsin B (D) and cathepsin D (E) were determined as described in the Materials and Methods section; 50 μM E64 (inhibitor of cathepsin B) and 50 μM pepstatin A (inhibitor of cathepsin D) were used as positive controls. All experiments were performed at least three times with the same results.

In order to identify compounds with USP7 inhibitory activity, using the Ub-AMC protease assay, we screened a small homemade compound library, which is composed of some FDA approved drugs, compounds entered in clinical trials and active natural compounds (Supplementary Table S1). The results showed that CDDO-Me (Figure 1A) markedly inhibited the USP7-mediated cleavage of GST-UBA52 (Figure 1B) in an *in vitro* gel-based assay. The IC_50_ of CDDO-Me for USP7 inhibition was 14.08 μM (Figure 1C). USP7 belongs to cysteine protease, which including palpain-like proteases (such as cathepsin B), caspase-like enzymes and deubiquitinating enzymes. To see whether CDDO-Me affects other cysteine protease, we measured its effect on cathepsin B and cathepsin D. Even at a concentration of 100 μM, CDDO-Me could not significantly inhibit the activity of cathepsin B and cathepsin D (Figure 1D, 1E). By contrast, E64 and pepstatin A, which are known inhibitors of cathepsin B and cathepsin D, markedly inhibited the activities of cathepsin B and cathepsin D (Figure 1D, 1E). Moreover, we examined the effect of CDDO-Me on other deubiquitiating enzymes with the similar structure to USP7. Interestingly, CDDO-Me also has inhibitory activity against USP2 with IC_50_ at 22.33 μM (Supplementary Figure S1). Together, these data show that CDDO-Me could inhibit USP7 activity *in vitro*, but does not inhibit palpain-like enzymes.

### CDDO-Me inhibits USP7 activity independent of the Michael acceptor in the A ring

**Figure 2:**
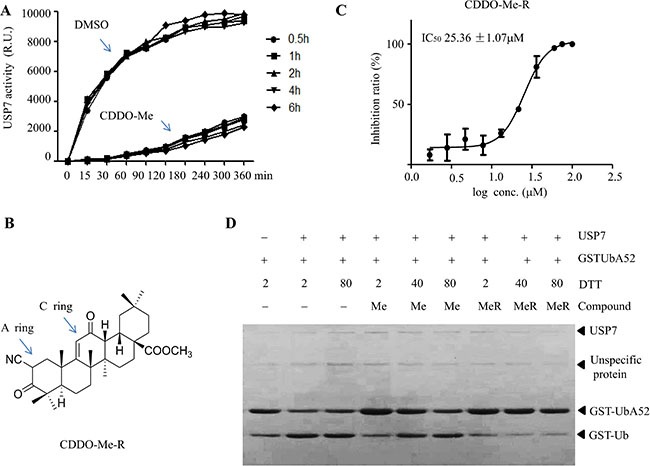
Reduced CDDO-Me inhibits USP7 (**A**) Time course of the inhibitory effect of CDDO-Me on USP7. USP7 was pre-incubated for different time periods with DMSO or CDDO-Me before initiating the enzymatic reaction by adding the Ub-AMC substrate (300 nM), and the activity of USP7 was measured. (**B**) Chemical structure of reduced CDDO-Me (CDDO-Me^R^). (**C**) The inhibitory effect of CDDO-Me^R^ on USP7 activity was assessed by a gel-based assay and IC_50_ was determined. (**D**) CDDO-Me (Me) and CDDO-Me^R^ (Me^R^) were pre-incubated with different concentrations of DTT, after which their inhibitory effect on USP7 was determined by a gel-based assay. All experiments were performed at least three times with the same results.

We next tried to determine the mode of action of CDDO-Me on USP7. CDDO-Me has two electrophilic Michael acceptor sites in the A and C rings. CDDO-Me can interact with proteins containing structurally available redox-sensitive cysteine residues such as IKK, STAT3 [24]. Given that USP7 is a cysteine protein, we hypothesized that CDDO-Me may covalently bind to USP7 and inhibit its activity in an irreversible manner. Unexpectedly, our results showed that CDDO-Me inhibited USP7 activity in a reversible manner (Figure 2A). Therefore, we suspected that the two Michael acceptor sites may not be necessary for the inhibitory effect of CDDO-Me. To address this, we attempted to reduce the double bonds in the A and C rings of CDDO-Me. However, we could only reduce the double bond in the A ring could be (CDDO-Me^R^) (Figure 2B). Interestingly, CDDO-Me^R^ inhibited the USP7 activity at concentrations similar to that of CDDO-Me (Figure 2C). Moreover, preincubation with dithiothreitol (DTT) at higher concentrations (40–80 mM) abrogated the activity of CDDO-Me but not that of CDDO-Me^R^ (Figure 2D). These data suggest that CDDO-Me inhibits USP7 activity via a mechanism independent of the presence of the Michael acceptor site in the A ring.

The binding mode between USP7 and CDDO-Me was further explored by molecular docking. The predicted USP7-CDDO-Me complex showed that the small molecule was bound to a narrow pocket near the catalytic cleft (Supplementary Figure S2A). CDDO-Me fits very well in this small pocket (Supplementary Figure S2B), thereby leading to its stable binding with USP7. The cyano group and the nearby carbonyl group of the molecule formed hydrogen bonds with the Gln297 and Asp295 residues of USP7, respectively. In addition, CDDO-Me had hydrophobic interactions with the Met292, Tyr465, Phe409 and Tyr411 residues. In the USP7-ubiquitin complex structure (PDB code: 1NBF), we found that the same narrow pocket was occupied by the ubiquitin C- terminus (Supplementary Figure S3). These results suggest that the inhibition mechanism of CDDO-Me may be explained by its displacement of the ubiquitin C terminus while binding with USP7 [25–26]. However, further experimental data, such as crystal structures, are necessary to confirm this docking hypothesis.

### USP7 is expressed at a higher level in ovarian cancer cells

**Figure 3:**
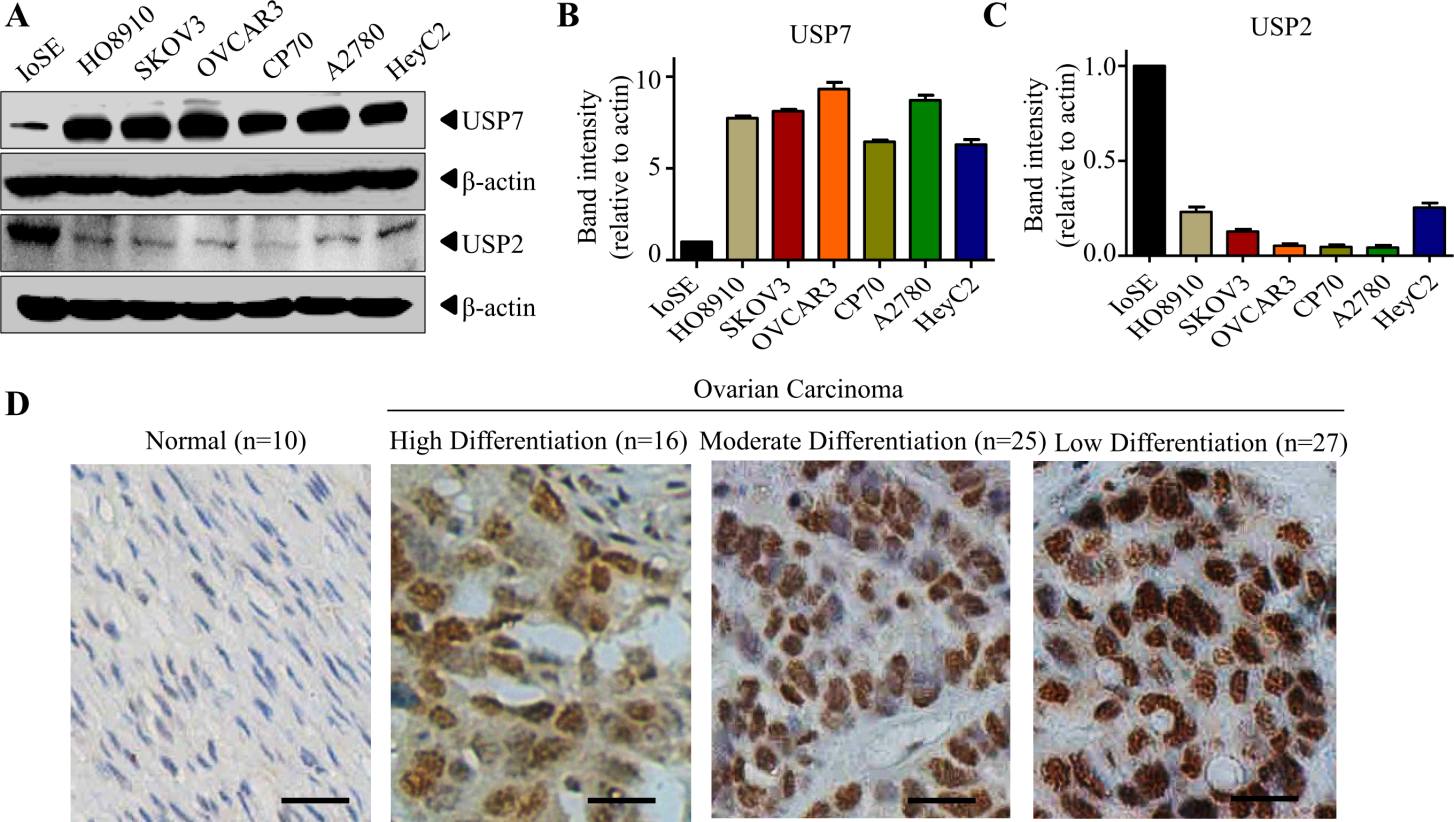
USP7 is elevated in ovarian cancer cells (**A**–**C**) Cell lysate from ovarian cancer cell lines and normal ovarian epithelial IOSE cells were subjected to Western blot analysis, and the indicated protein was examined. The intensity of the bands was quantified using the Quantity One software (B for USP7, C for USP2). (**D**) Ovarian cancer tissues from non-neoplastic, serous carcinoma, mucinous adenocarcinoma and endometrioid carcinoma were stained with the isotype control or anti-USP7 antibodies. Brown (DAB) coloured cells showed USP7 staining. The micrograph (original magnification, 200×) shows representative tissues with normal phenotype, high differentiation carcinoma, moderate differentiation carcinoma and low differentiation carcinoma of ovarian cancer based on USP7 expression. Scale bar = 50 μm.

CDDO-Me has been found to be effective in ovarian cancers on tumor cell death [22–23, 27]. As CDDO-Me could directly inhibit USP7 activity *in vitro*, we hypothesized that USP7 may be a novel target of CDDO-Me in ovarian cancer cells. Although overexpression of USP7 has been observed in several types of cancer cells, its association with ovarian cancer cells is unknown. Therefore, we first examined the expression of USP7 in ovarian cell lines. Compared with the non-tumorigenic immortalized ovarian surface epithelial (IOSE) cells, several ovarian cancer cell lines exhibit higher USP7 expression (Figure 3A, 3B). However, the expression of USP2 is lower in these cell lines (Figure 3A, 3C). Subsequently, we examined the expression of USP7 in ovarian cancer samples by immunohistochemistry. Ovarian cancer tissues from non-neoplastic (*n* = 10), serous carcinoma (*n* = 46), mucinous adenocarcinoma (*n* = 15) and endometrioid carcinoma (*n* = 7) were stained with anti-USP7 antibodies. As shown in Figure 3D, USP7 was localized to the nucleus of the respective tissues. In general, ovarian cancer tissues expressed significantly higher levels of USP7 compared with non-neoplastic tissue (*p* < 0.001, Table 1). Moreover, an inverse relationship was observed between the degree of differentiation and USP7 expression: the lower the degree of differentiation, the higher the USP7 expression (Figure 3D, Table 1, *p* < 0.001). These data indicate that USP7 is expressed at higher levels in ovarian cancer cells than in normal cells.

**Table 1:** Clinicopathological features of ovarian tissue with regard to the relative expression of USP7 protein

Variables	Numbers of specimens	High expression	*p* value
Normal ovary	10	0/10 (0%)	
Ovarian carcinoma	68	62/68 (91.2%)	*p* < 0.001
Serous adenocarcinoma	46	43/46 (93.5%)	*p* < 0.001
Mucous adenocarcinoma	15	12/15 (80%)	*p* < 0.001
Endometrioid carcinoma	7	7/7 (100%)	*p* < 0.001
**Differentiation**			*p* < 0.001
High	16	2/16 (12.5%)	
Moderate/Low	52	48/52 (92.3%)	

### Knockdown of USP7 inhibits proliferation of ovarian cancer cells

**Figure 4:**
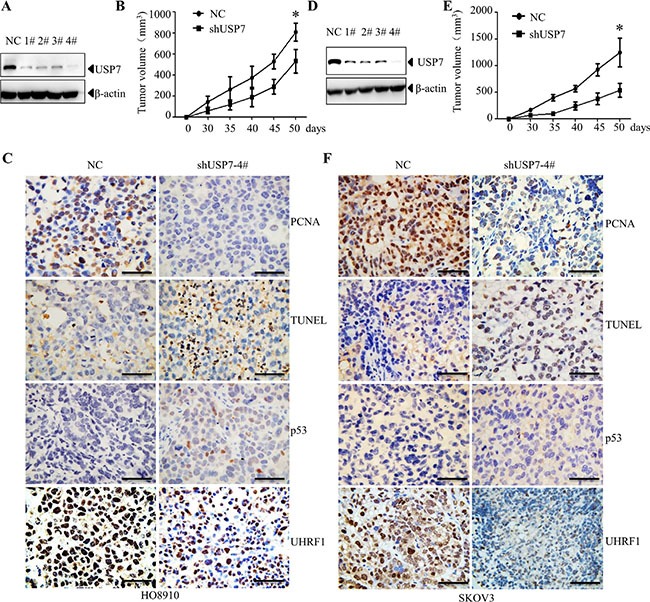
Knockdown of USP7 inhibits growth of ovarian xenograft tumor USP7-specific shRNA and non-specific shRNA (NC) were transfected into H08910 (**A**) and SKOV3 (**D**) cells, respectively. The expression level of USP7 was determined by Western blot analysis. USP7 knockdown cells (2 × 10^6^) and control cells (2 × 10^6^) were subcutaneously implanted into nude mice. The sizes of tumor were monitored for the indicated time periods in H08910 (**B**) and SKOV3 (E) cells. *p < 0.05. Representative images showing PCNA, TUNEL, p53 and UHRF1 expression in H08910 (**C**) and SKOV3 (**F**) cells. Scale bars=50 μm.

To determine the role of USP7 in ovarian cancer cells, USP7 was stably knocked down in HO8910 and SKOV3 cells (Figure 4A, 4D). The vector-transfected control cells and USP7 knockdown cells were subcutaneously inoculated into nude mice, respectively. As shown in Figure 4B and 4E, compared with the non-specific shRNA (NC) transfected cells, knockdown of USP7 (shUSP7) significantly inhibited tumor growth in nude mice. Consistent with these observations, the percentage of PCNA-positive cells significantly decreased, whereas the percentage of TUNEL-positive cells significantly increased in USP7-silenced cells (Figure 4C, 4F). These findings indicate the inhibition of proliferation and increased cell death occurred upon USP7 knockdown. Given that p53 is expressed in HO8910 cells but not in SKOV3 cells [28–30], we observed an increase in p53 in the USP7-silenced HO8910 cells (Figure 4C, 4F). Moreover, decrease of UHRF1 was observed in USP7 silenced cells (Figure 4C, 4F). These data suggest that USP7 plays an important role in the proliferation and survival of ovarian cancer cells.

### CDDO-Me interacts with and inhibits the activity of USP7 in ovarian cancer cells

**Figure 5:**
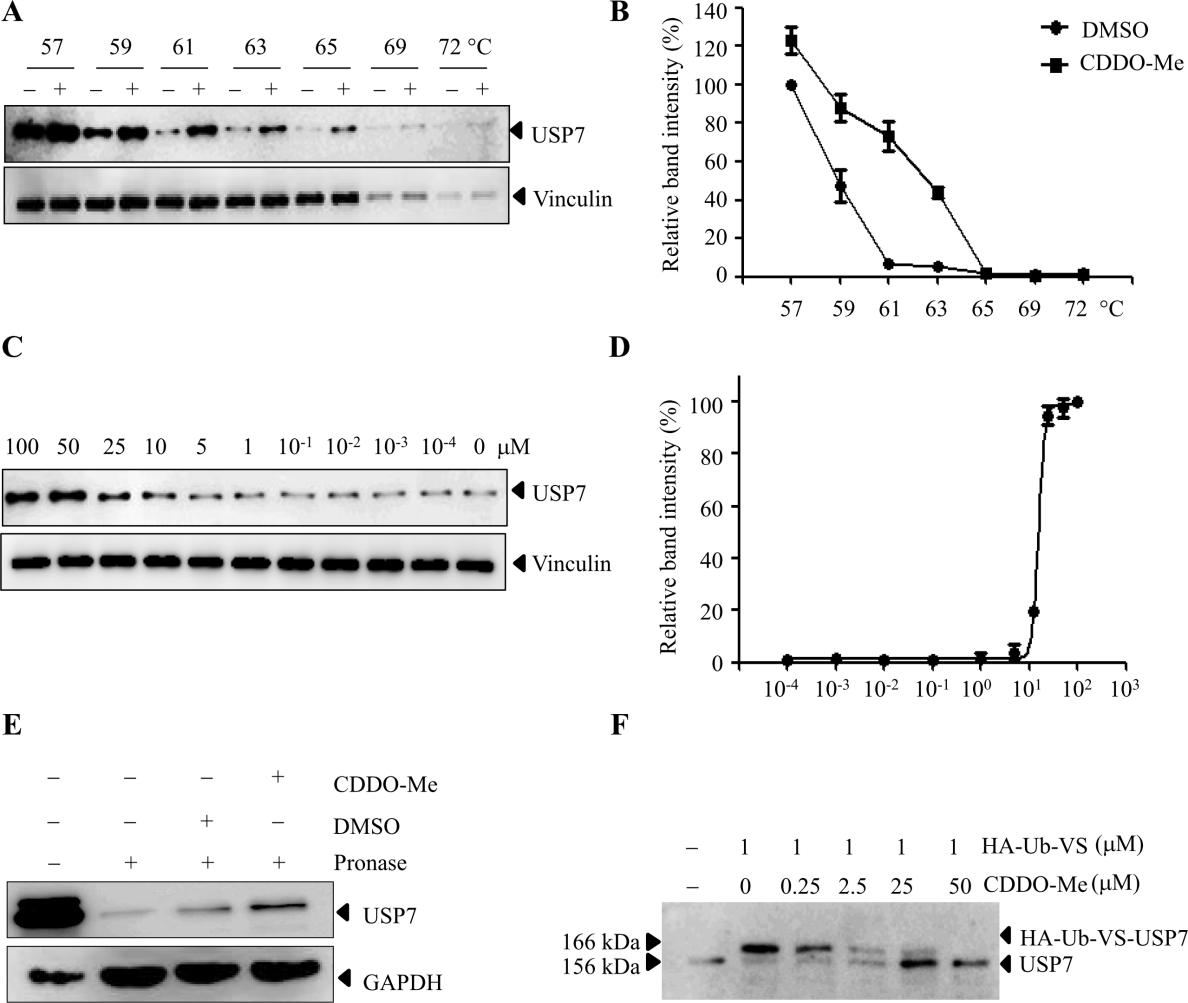
CDDO-Me interacts with USP7 in cells (**A**–**D**) CETSA was performed on HO8910 cells as described in the Materials and Methods section. The stabilizing effects of CDDO-Me on USP7 and vinculin at different temperatures (A) and different doses (C) were evaluated by Western blot analysis. The intensity of the USP7 bands was quantified using the Quantity One software (B, D). (**E**) DARTS was performed on HO8910 cells as described in the Materials and Methods section. Protection of USP7 from proteolysis by CDDO-Me was evaluated by Western blot analysis. (**F**) HO8910 cell lysate was pre-incubated with different doses of CDDO-Me for 30 min and then treated with HA-Ub-VS for another 30 min. The lysates were subjected to Western blot analysis with the antibody against USP7. All experiments were performed at least three times with the same results.

Based on the above results, we hypothesized that CDDO-Me may directly inhibit USP7 in ovarian cancer cells. To this end, cellular thermal shift assay (CETSA) was used in H08910 cells. CETSA is a newly-developed method to evaluate drug binding to target proteins in cells and tissue samples, which is based on the biophysical principle of ligand-induced thermal stabilization of target proteins [31–32]. Compared to DMSO-treated cell lysate, CDDO-Me markedly increased the thermal stability of USP7 at temperatures examined (Figure 5A, 5B). We also tested whether USP7 stability during heating depended on the dose of CDDO-Me. As shown in Figure 5C and 5D, USP7 accumulation markedly increased as CDDO-Me concentration increased. As a negative control, we demonstrated that CDDO-Me did not increase the stability of vinculin in cells. These data suggest that CDDO-Me directly interacts with USP7 in cells. Consistent with the CETSA results, the drug affinity responsive target stability (DARTS) assay [33] also showed that the presence of CDDO-Me partially prevents pronase-mediated digestion of USP7 (Figure 5E). Furthermore, CDDO-Me could compete with the binding of hemagglutinin [HA]-tagged ubiquitin-vinyl sulfone (HA-Ub-VS), a DUBs active site directed probe, to USP7 in a dose-dependent manner (Figure 5F). Taken together, these results confirm that CDDO-Me interacts with USP7 in cells.

**Figure 6:**
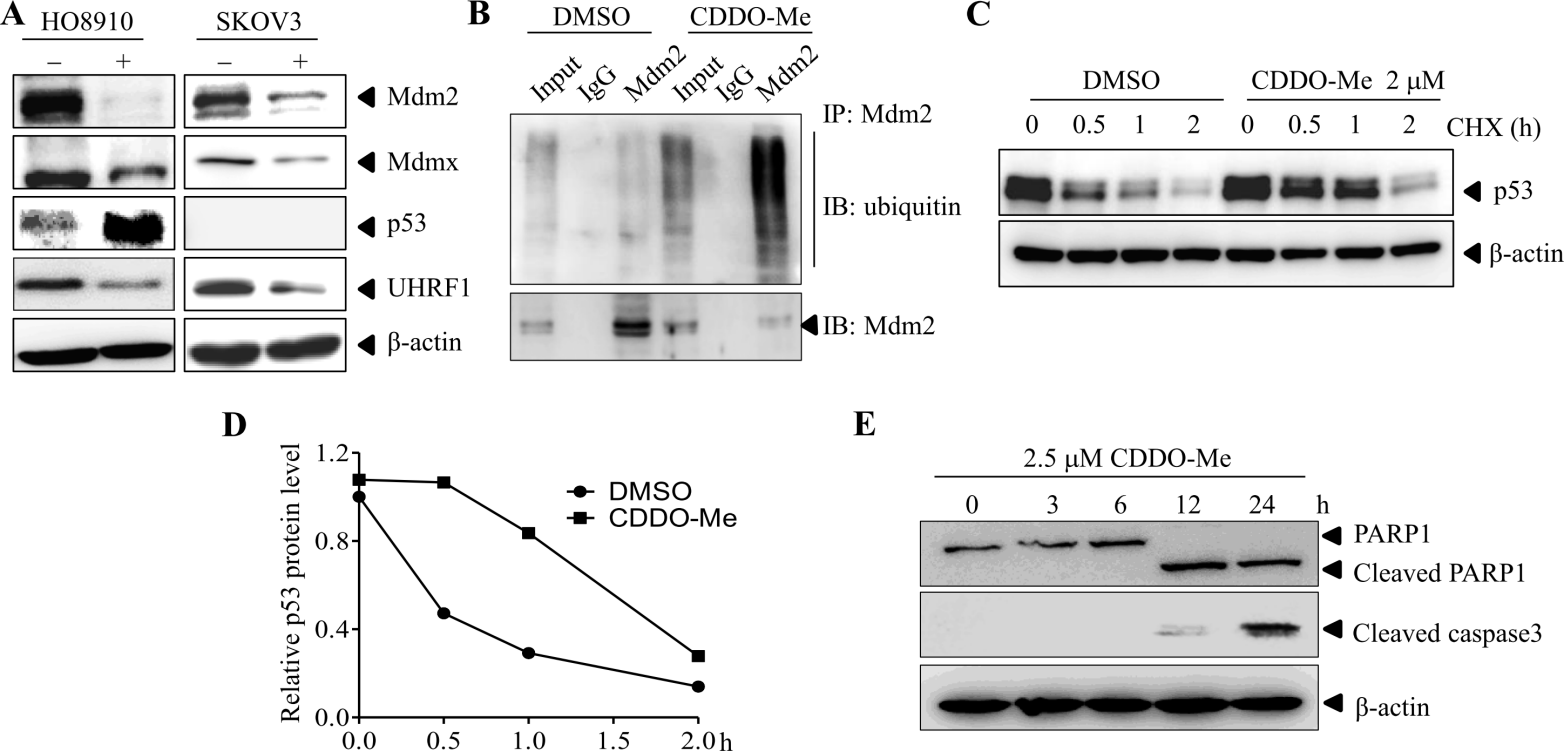
CDDO-Me inhibits USP7 activity in ovarian cancer cells (**A**) HO8910 and SKOV3 cells were treated with CDDO-Me for the indicated durations and protein levels were examined by Western blot analysis. (**B**) MDM2 was immunoprecipitated from CDDO-Me- and DMSO-treated HO8910 cells. The indicated proteins were examined by Western blot analysis. (**C**) In the presence of cycloheximide (CHX), HO8910 cells were treated with CDDO-Me or DMSO for different time periods. The protein level of p53 was determined by Western blot analysis with β-actin as a loading control. (**D**) The intensity of the p53 bands was quantified by the Quantity One software and normalized to β-actin. (**E**) HO8910 cells were treated with CDDO-Me for different time periods and the indicated proteins were examined by Western blot analysis. All experiments were performed at least three times with the same results.

Inhibition of USP7 may lead to changes in its substrates such as MDM2, MDMX, P53 and UHRF1. To test this hypothesis, HO8910 and SKOV3 cells were treated with CDDO-Me for different time periods, and the protein levels of MDM2, MDMX, P53 and UHRF1 were determined by western blotting. As shown in Figure 6A, CDDO-Me treatment led to decreased levels of MDM2, MDMX and UHRF1. By contrast, p53 level markedly increased. Consistent with the idea that CDDO-Me inhibits the deubiquitinase activity of USP7, the amount of ubiquitinated MDM2 markedly increased after CDDO-Me treatment (Figure 6B). In addition, the half-life of p53 increased from 0.5 h to 1–2 h (Figure 6C, 6D). Accompanied with the inhibition of USP7, CDDO-Me treatment led to activation of caspase-3 and cleavage of PARP1, indicating induction of apoptosis (Figure 6E). Taken together, these data suggest that CDDO-Me inhibits USP7 activity in ovarian cancer cells.

### CDDO-Me inhibits USP7 *in vivo*

**Figure 7:**
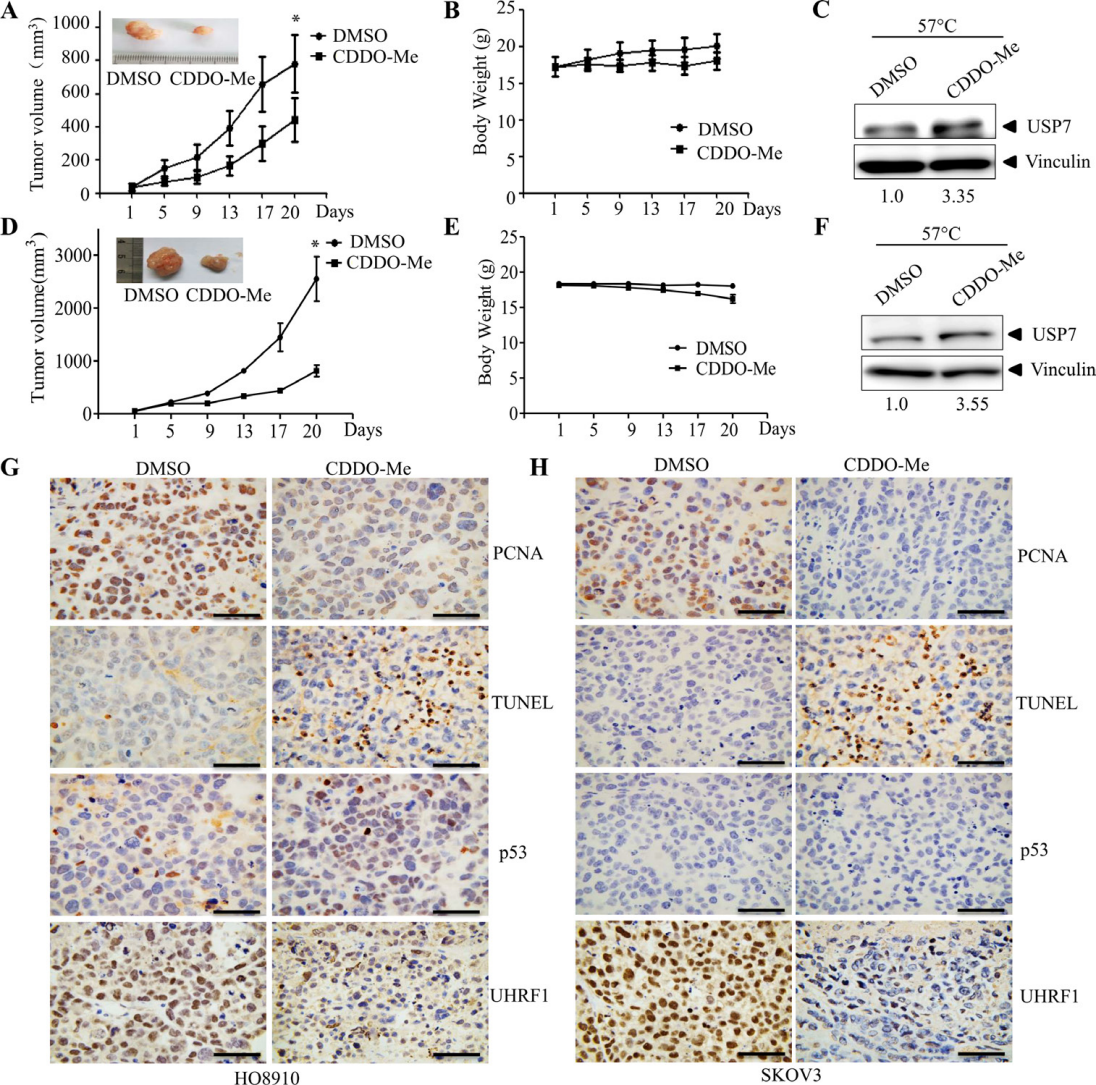
CDDO-Me inhibits growth of ovarian cancer cells *in vivo* and interacts with USP7 in tissues HO8910 cells (5 × 10^6^) or SKOV3 (5 × 10^6^) were subcutaneously transplanted into nude mice. When tumors became palpable, CDDO-Me (20 mg/kg) and vehicle were intraperitoneally injected. The sizes of tumor were monitored for the indicated number of days (**A**) for HO8910, (**D**) for SKOV3. *p < 0.05. Body weights of the animals were monitored for the indicated number of days (**B**) for HO8910, (**E**) for SKOV3. Interaction of CDDO-Me with USP7 in tumor tissues was examined by CETSA as described in the Material and Methods section (**C**) for HO8910, (**F**) for SKOV3. The signal intensity of USP7 proteins against vinculin was quantified by Quantity One. Representative images showing PCNA, TUNEL, p53 and UHRF1 expression (**G**) for HO8910, (**H**) for SKOV3. Scale bar = 50 μm.

Finally, we investigated the *in vivo* efficacy of CDDO-Me in the HO8910 and SKOV3 xenograft model. Intraperitoneal injection of CDDO-Me inhibited HO8910 and SKOV3 tumor growth (Figure 7A, 7D). Consistent with previous reports [34], mild weight loss was observed in the CDDO-Me treatment group (Figure 7B, 7E). CDDO-Me also decreased the proliferation and increased the cell death of tumor cells, as assessed by PCNA and TUNEL staining (Figure 7G, 7H). Moreover, CETSA assays showed that CDDO-Me treatment increase the thermal stability of USP7 in tumor tissues, indicating the interaction of CDDO-Me and USP7 (Figure 7C, 7F). Furthermore, CDDO-Me increased p53 protein levels in HO8910 cells but decreased UHRF1 in HO8910 and SKOV3 cells, indicating the inhibition of USP7 activity (Figure 7G, 7H). Together, these data indicate that CDDO-Me inhibits USP7 activity in tumor tissues.

## DISCUSSION

The synthetic oleanane triterpenoid CDDO-Me is a multifunctional compound that has been studied in phase I and phase II clinical trials for treating haematological malignancies and solid tumors. In this study, we demonstrated that CDDO-Me directly inhibits USP7 activity *in vitro* and *in vivo*. Using CDDO-Me as a probe, we found that USP7 is overexpressed in ovarian cancer cells. Targeting USP7 may thus contribute to the antiproliferative and pro-apoptotic activities of CDDO-Me.

Recent studies have reported elevated levels of USP7 in prostate cancer and gliomas [35–36]. USP7 is also implicated in modulating tumor growth and apoptosis in a colon carcinoma xenograft model [37]. Several USP7 inhibitors have shown potent efficacy against tumor cells [38]. Therefore, USP7 is an attractive drug target for cancer therapy. In this study, we have shown that USP7 is a direct novel target of CDDO-Me based on following evidence: (a) the gel-based assay showed that CDDO-Me could inhibit USP7 activity *in vitro*; (b) CETSA and DARTS analyses demonstrated that CDDO-Me directly interacts with USP7 in cells; (c) CDDO-Me could compete with the binding of USP7 to HA-Ub-VS in cells; (d) consistent with the inhibition of USP7 activity, treatment with CDDO-Me decreased the levels of USP7 substrates, such as MDM2, MDMX, and UHRF1.

CDDO-Me has two α, β-unsaturated carbonyl moieties. The proposed mechanism underlying the anti-cancer effect of CDDO-Me is the formation of Michael adducts between CDDO-Me and reactive nucleophiles, such as free thiols, on target proteins [24]. USP7 is a cysteine protease. Initially, we hypothesized that CDDO-Memay covalently bind to the active cysteine in USP7. However, reduced CDDO-Me also inhibits the activity of USP7 with similar potency compared to CDDO-Me. Therefore, CDDO-Me is unlikely to inhibit USP7 via an α, β-unsaturated carbonyl moiety-dependent pathway. Our docking studies showed that CDDO-Me fits well in the ubiquitin-binding pocket. This model may explain why DTT could abrogate the effect of CDDO-Me. After reacting with DTT, CDDO-Me may not fit as well in the same pocket. As expected, the binding of HA-Ub-VS with USP7 could be abrogated by pre-incubation of CDDO-Me with USP7 [39]. This result may also provide explanation for the inhibitory activity of CDDO-Me against USP2. Currently, several USP7 inhibitors, such as HBX41,108, HBX19,818 and P5091, are available [40–41]. Similar to HBX41,108, CDDO-Me is a non-covalent inhibitor of USP7. However, the binding pocket of CDDO-Me is similar to that of HBX19,818. That is, CDDO-Me occupies the ubiquitin-binding pocket of USP7 by interacting with Asp295 and Gln297. Both of these residues are located at the entrance of the ubiquitin-binding pocket. Therefore, CDDO-Me may represent a novel non-covalent inhibitor of USP7 by binding to the C-terminus of ubiquitin.

Although CDDO-Me can inhibit the activity of USP7 *in vitro*, an important question is whether CDDO-Me indeed interacts with USP7 in cells. We used CETSA to evaluate the interaction between CDDO-Me and USP7. CETSA is a newly-developed method monitor and quantify the extent to which a drug candidate reaches and directly binds to a protein target of interest within a cell. The CETSA methodology is based on the thermodynamic stabilization observed for individual proteins as a result of ligand binding. Compared with other methods that confirm the interaction of small molecule compounds with proteins, CETSA is more feasible and can directly measure whether a drug molecule reaches its targets in cells [31–32, 42]. Using this method, we demonstrated that CDDO-Me interacts with USP7 in cells. In addition, CDDO-Me could protect USP7 from protease-mediated protein cleavage during DARTS assay. These data indicate that CDDO-Me could interact with USP7 in its relevant cellular context. Consistent with the inhibition of USP7 activity in cells, CDDO-Me treatment led to the decrease of its substrates including MDM2, UHRF1.

CDDO-Me can effectively inhibit the growth of ovarian cancer cells *in vitro* [22–23, 27]. Here we found that CDDO-Me can inhibit USP7 activity. Therefore, we suspected that USP7 may be a target of CDDO-Me in ovarian cancer cells. Interestingly, several tumor suppressor genes, including p53, PTEN, RB1, BRCA1 and UHRF1 are either mutated or dysregulated in ovarian cancer. Evidence shows that all of these proteins can be regulated by USP7. However, the role of USP7 in ovarian cancer remains unclear. We provide evidence that USP7 is expressed at higher level in ovarian cancer cell lines and primary ovarian cancer cells. In contrast, lower level of USP2 was observed in ovarian cancer cells. Interestingly, the expression level of USP7 is inversely related to the degree of differentiation of ovarian cancer cells. Moreover, knockdown of USP7 significantly inhibits the growth of H08910 (p53-wild) and SKOV3 (p53-null) cells *in vitro* and *in vivo*, suggesting that USP7 plays an important role in the proliferation of ovarian cancer cells. And the growth inhibition effect of USP7 knockdown does not rely on p53. UHRF1, an USP7 substrate, has been observed in many cancer tissues such as breast, bladder, kidney, lung, prostate, cervical and pancreatic cancers [43–46]. It functions as an oncogene in the tumorigenesis and cancer progression [47–48]. Recently, UHRF1 was found to be a new oncogenic factor in ovarian cancer as knockdown of UHRF1 inhibits ovarian cancer cell proliferation [49]. As CDDO-Me decreases the expression of UHRF1, UHRF1 downregulation may contribute to CDDO-Me-induced cell growth inhibition.

In conclusion, using CDDO-Me as a probe, we identified USP7 as a novel target in ovarian cancer. Targeting USP7 may represent a novel strategy to treat ovarian cancer. Because CDDO-Me and its related synthetic triterpenoids have entered clinical trials as promising preventive and therapeutic agents for cancer and chronic inflammatory diseases, development of novel USP7-selective compounds based on the CDDO-Me scaffold warrant further investigation.

## MATERIALS AND METHODS

### Cell Culture

The human epithelial ovarian cancer cells SKOV3, OVCAR3, A2780, A2780/CP70, and HeyC2 cell lines were purchased from the American Type Culture Collection (ATCC, Manassas, VA). HO8910 was obtained from the Cell Bank of the Chinese Academy of Sciences (Shanghai, China). Cells were cultured in RPMI-1640 (Gibco, Foster City, CA) supplemented with 10% (w/v) fetal bovine serum (FBS; Gibco) and 1% penicillin–streptomycin (Gibco). Human nontumorigenic IOSE were gifts from Professor M. W.-Y. Chan (National Chung Cheng University, Taiwan). Human embryonic kidney 293T (HEK293T) cells were cultured in Dulbecco's Modified Eagle's Medium (Gibco) with 10% FBS and 1% penicillin–streptomycin. All cell lines were maintained at 37°C in a humidified atmosphere with 5% CO_2_.

### Plasmid construction

Human UBA52 including a N-terminally fused GST tag was constructed by PCR amplification with the forward primer 5′-TCGCGGATCCAT GCAGATCTTTGTGAAGACCCTC-3′ and the reverse primer 5′-TCCGCTCGAGTTATTTGACCTTCTT CTTGGGACG-3′. The PCR products were inserted in pGEX-6P1. All the constructs were verified by DNA sequencing.

### Purification of GST-UBA52

The pET28a(+)-GST-UBA52 plasmid was transfected into *Escherichia coli* BL21. The expression of GST-UBA52 was induced with 0.5 mM isopropyl-D-thiogalactoside (IPTG) at 16°C for 12 h. Cell pellets were resuspended in lysis buffer (300 mM NaCl; 50 mM PBS, pH 8.0; 10 mM imidazole; 10 mM β-mercaptoacetic ethanol; 10% glycerol) and sonicated. The His-tagged proteins were purified with Ni-NTA–agarose and eluted with 250 mM imidazole in 300 mM NaCl, 50 mM PBS (pH 8.0), 10 mM β-mercaptoacetic ethanol and 10% glycerol.

### Gel-Based USP7 activity assay *In Vitro*

USP7 (80 nM) was incubated with compounds for 10 min at 37°C in reaction buffer (50 mM Tris-HCl, pH 8.0; 20 mM NaCl; 2 mM DTT). Subsequently, GST-UBA52 (3.92 mM, final concentration) was added. The mixture was incubated for another 45 min at 37°C. The reaction was terminated by adding the loading buffer and boiling on the heat block. The proteins were separated by 12% sodium dodecyl sulphate–polyacrylamide gel electrophoresis (SDS-PAGE) and visualised with Coomassie brilliant blue (G250).

### Cathepsin B activity assay

The effect of CDDO-Me on the activity was evaluated by the cathepsin B activity assay kit (BioVision Inc., Mountain View, CA). Briefly, 5 × 10^6^ SKOV3 cells were collected and lysed in 500 μL lysis buffer for 10 min on ice. After centrifugation (15000 rpm, 4°C, 10 min), the supernatant was pre-incubated with 50 μM E64 (an inhibitor of cathepsin B; Calbiochem); DMSO; and 50 or 100 μM CDDO-Me, respectively, at 37°C for 30 min. The substrate RR-AFC was added into the reaction systems, which were incubated for another 1.5 h at 37°C. The fluorescence of AFC was determined by the Synergy H4 Hybrid Microplate Reader with excitation at 400 nm and emission at 505 nm.

### Cathepsin D activity assay

The effect of CDDO-Me on the activity was evaluated by the cathepsin D activity assay kit (BioVision Inc.). Briefly, 5 × 10^6^ SKOV3 cells were collected and lysed in 500 μL lysis buffer for 10 min on ice. After centrifugation (15 000 rpm, 4°C, 10 min), the supernatant was pre-incubated with 50 μM pepstatin A (inhibitor of cathepsin D); DMSO; and 50 or 100 μM CDDO-Me at 37°C for 30 min. The substrate GKPILFFRLK (Dnp)-D-R-NH_2_-labelled with 7-methoxycoumarin-4-ylacetyl (MCA) was added into the reaction systems, which were incubated for 1.5 h at 37°C. The fluorescence of MCA was determined by the Synergy H4 Hybrid Microplate Reader with excitation at 328 nm and emission at 460 nm.

### Ub-AMC protease assay

Ub-AMC hydrolysis assays were performed at 25°C in the assay buffer (50 mM Tris/HCl, pH 7.5; 150 mM NaCl; 2 mM EDTA; 2 mM DTT), which was supplemented with 1 mg/mL bovine serum albumin (Roche). The recombinant USP7 (20 nM) was pre-incubated with DMSO (%) or the respective compounds (50 μM) for 30 min; the enzymatic reaction was initiated by adding the Ub-AMC substrate (300 nM). The reaction mixture was incubated at room temperature for 1 h before the reaction was stopped by adding acetic acid (100 mM). The fluorescence emission intensity was measured on a Synergy H4 Hybrid Microplate Reader, with a 368 nm/467 nm filter pair and a 455 nm cut-off.

### Synthesis of reduced CDDO-Me

A mixture of CDDO-Me (110 mg, 0.2 mmol) and 5% Pd/C (5 mg) in MeOH (10 mL) was stirred at room temperature under H_2_ at atmospheric pressure for 12 h. The reaction mixture was filtered. The insoluble substance was washed with MeOH (10 mL, three times). The filtrate was concentrated in vacuo to give CDDO-Me-R as a white solid (103 mg, 94%).

^1^H NMR (500 MHz, CDCl_3_, δ): 5.74 (m, 1H), 3.69 (s, 3H), 3.04 (m, 1H), 2.91 (m, 1H), 2.34 (s, 1H), 2.03 (s, 1H), 1.87 (s, 2H), 1.56 (m, 14H), 1.26 (s, 6H), 1.21 (m, 6H), 1.13 (m, 3H), 1.00 (s, 6H).

MS (electrospray): *m/z* 508.3425 ([M+H]^+^, calculated 508.3421).

### Western blot analysis

Cells were washed with PBS and lysed with the lysis buffer (50 mM Tris-HCl, pH 6.8; 100 mM DTT, 2% SDS; 10% glycerol). Cell lysates were centrifuged at 20,000 × *g* for 10 min; the proteins in the supernatants were quantified. Protein extracts were equally loaded on 8%–12% gels for SDS-PAGE. The separated proteins were transferred onto nitrocellulose membranes (Bio-Rad). The blots were stained with 0.2% Ponceau S red to ensure equal protein loading. After blocking with 5% non-fat milk in PBS, the membranes were probed with antibodies against Flag (Sigma-Aldrich), HA (Sigma-Aldrich), β-tubulin (Sigma-Aldrich), β-actin (Calbiochem), P53, Mdm2, caspase-3 and PAPR1. The signals were detected with a chemiluminescence phototope-HRP kit (Cell Signalling) according to the manufacturer's instructions. When necessary, blots were stripped and reprobed with anti-β-actin or β-tubulin antibody as internal control. All experiments were performed three times with similar results.

### Cellular thermal shift assay

HO8910 and SKOV3 cells were harvested and washed with PBS. The cells were diluted in PBS supplemented with a complete protease inhibitor cocktail. The cell suspensions were freeze-thawed three times with liquid nitrogen. The soluble fraction (lysate) was separated from the cell debris by centrifugation at 20,000 × *g* for 20 min at 4°C. The cell lysates were diluted with PBS and divided into two aliquots. One aliquot was treated with DMSO; the other aliquot was mixed with the diluent of CDDO-Me. After 10–30 min of incubation at room temperature, the respective lysates were divided into smaller (50 μL) aliquots and individually heated at different temperatures for 3 min (Veriti thermal cycler, Applied Biosystems/Life Technologies), followed by cooling for 3 min at room temperature. The appropriate temperatures were determined in preliminary CETSA experiments (data not shown). The heated lysates were centrifuged at 20,000 × *g* for 20 min at 4°C to separate the soluble fractions from the precipitates. The supernatants were transferred to fresh microtubes before SDS-PAGE and Western blot analysis.

### Drug affinity responsive target stability

DARTS is a general methodology for identifying and studying protein–ligand interaction; this method was employed to evaluate the CDDO-Me–USP7 interaction. HO8910 cell pellets were lysed in standard Triton X-100 lysis buffer (50 mM Tris-HCl, pH 7.5; 200 mM NaCl; 0.5% Triton X-100; 10% glycerol; 1 mM DTT), which was supplemented with protease inhibitor cocktails (Sigma). The cell lysates were incubated with drugs or vehicle in room temperature for 50 min. The above mentioned mixture continued to be digested by pronase (10 mg/mL stock solutions in water-based aliquots stored at −20°C) at appropriate ratios dissolved in TNC buffer (50 mM Tris-HCl, pH 8.0; 50 mM NaCl; 10 mM CaCl_2_) for 30 min. The reaction was stopped by the addition of concentrated SDS-PAGE loading buffer to a final concentration of 1×, well mixed and immediately boiled. Finally, the samples were subjected to Western blot analysis.

### Molecular docking

Molecular docking was performed with the AutoDock4.2 software. The X-ray structure of the USP7 catalytic domain (PDB ID: 4M5W) was retrieved from the Protein Data Bank (http://www.rcsb.org/pdb) for docking calculation. Water and bromide ions were all removed. To prepare for both the protein and small molecule, all hydrogen atoms were added first, and the Gasteiger charges were computed before the non-polar hydrogen atoms were merged. The active site was defined in AutoGrid4 by a grid box as large as 70 × 70 × 70 points with a grid spacing of 0.375 Å. The box was centred on the Tyr514 residue in the crystal structure of USP7. The protein was considered rigid for the docking study. The docking parameters were set as ga_pop_size = 150 (number of individuals in population) and ga_run = 100 (number of dockings that were performed). Default values in the software were set for other parameters. Protein–ligand interactions were accounted by the Lamarckian genetic algorithm. Finally, the conformation was selected according to the predicted binding free energy.

### Tissue microarrays and immunohistochemistry

An ovarian cancer tissue array (US Biomax, Inc., Rockville, MD) was used for this study; the array contained ovarian tumors from 68 patients and 10 normal tissue samples. Deparaffinisation and antigen retrieval were accomplished with the Trilogy solution (Cell Marque, Rocklin, CA). The heating/pressure was supplied by a conventional pressure cooker. Endogenous peroxidase activity was inhibited by 0.3% hydrogen peroxide. Non-specific interactions were blocked by normal goat serum. The USP7 antibody (ab26083, abcam) was diluted in PBS at 1:800 and incubated at 4°C overnight. The bound antibody was detected by the biotin-linked anti-rabbit secondary antibody and streptavidin-conjugated HRP enzymes in conjunction with the DAB chromagen. Tissue was counterstained with haematoxylin. Immunoreactivity was defined by the presence of discrete brownish chromogen deposits in the cells. A semi-quantitative scoring method of USP7 was used for immunohistochemical evaluation; the staining intensity and percentage positive-stained tumor cells were recorded. A staining index (with values from 0 to 9) was obtained from the intensity of USP7 staining multiplied by the proportion of immunopositive tumor cells. The staining intensity was scored as 0 (negative), 1 (weakly positive), 2 (moderate) or 3 (strong); the proportion of immunopositive tumor cells was scored as 1 (≤ 10%), 2 (10%–50%) or 3 (> 50%). The expression of USP7 in the epithelium of all 10 informative samples of normal ovarian samples was negative (0) or weak (1); thus, the staining index in the normal ovaries was determined to be 3 or lower. Therefore, we interpreted a staining index of 0 to 3 as the normal expression of USP7, whereas a staining index of 4 to 9 was interpreted as the overexpression of this protein. For each sample, the percentage of positive cells was recorded. The presence of more than 10% expression of the marker within the tumor cells was considered positive.

### RNA interference and transfection

Pairs of complementary oligonucleotides (Supplementary Table S2) were designed against USP7. The non-target control shRNA (NC) was synthesised by Sangon Biotech (Shanghai, China), annealed and ligated to the PSIREN-RetroQ Vector (Clontech Laboratories, Inc., CA, USA). The shRNA-carrying retroviruses, which were produced in 293T cells, were used to infect HO8910 and SKOV3 cells.

### Animal experiments

Female BALB/c nu/nu mice aged 4–6 weeks were kept under pathogen-free conditions according to the Shanghai Medical Experimental Animal Care guidelines. Animal protocols were approved by the Institutional Animal Care and Use Committee of Shanghai Jiao-Tong University School of Medicine.

Ovarian cancer HO8910 (2 × 10^6^) and SKOV3 (2 × 10^6^) cells with stable knockdown of USP7 were suspended in 0.2 mL of PBS and subcutaneously inoculated into the left flank of each mouse. Tumor sizes were measured with calipers; their volumes were calculated by the following standard formula: (width^2^ × length)/2; the body weight was measured every 2 days. After the animals were sacrificed, the xenograft tissues were immediately collected and stored at −80°C for further study.

For the drug treatment, HO8910 cells (5 × 10^6^) or SKOV3 (5 × 10^6^) suspended in 0.2 mL of PBS were subcutaneously inoculated into the left flank of each mouse. When tumors became palpable, mice were randomly divided into the control and treatment groups. Mice in the treatment group were intraperitoneally injected with CDDO-Me at 10 mg/kg daily for 20 days, whereas the control group was injected with DMSO. Tumor sizes were measured with calipers; their volumes were calculated by the following standard formula: (width^2^ × length)/2; the body weight was measured every 2 days. After the animals were sacrificed, the xenograft tissues were collected immediately and stored at −80°C for further study.

### Statistical analysis

The χ^2^ test was applied to test a possible association between the USP7 expression and the histological grade and type. A Student's unpaired two-tailed t test was used to assess the statistical significance of the tumor size. Values with *p* < 0.05 were considered statistically significant. All statistical analyses were performed with the SPSS software package (version 17.0, SPSS, Inc., Chicago, IL).

## SUPPLEMENTARY MATERIALS FIGURES AND TABLES





## References

[1] Bresalier RS, Kopetz S, Brenner DE (2015). Blood-based tests for colorectal cancer screening: do they threaten the survival of the FIT test?. Dig Dis Sci.

[2] Deng J, Wang L, Chen H, Hao J, Ni J, Chang L, Duan W, Graham P, Li Y (2016). Targeting epithelial-mesenchymal transition and cancer stem cells for chemoresistant ovarian cancer. Oncotarget.

[3] Huang L, Cronin KA, Johnson KA, Mariotto AB, Feuer EJ (2008). Improved survival time: what can survival cure models tell us about population-based survival improvements in late-stage colorectal, ovarian, and testicular cancer?. Cancer.

[4] Reyes-Turcu FE, Ventii KH, Wilkinson KD (2009). Regulation and cellular roles of ubiquitin-specific deubiquitinating enzymes. Annual review of biochemistry.

[5] Eletr ZM, Wilkinson KD (2014). Regulation of proteolysis by human deubiquitinating enzymes. Biochim Biophys Acta.

[6] Garcia-Santisteban I, Peters GJ, Giovannetti E, Rodriguez JA (2013). USP1 deubiquitinase: cellular functions, regulatory mechanisms and emerging potential as target in cancer therapy. Mol Cancer.

[7] Wang CL, Wang JY, Liu ZY, Ma XM, Wang XW, Jin H, Zhang XP, Fu D, Hou LJ, Lu YC (2014). Ubiquitin-specific protease 2a stabilizes MDM4 and facilitates the p53-mediated intrinsic apoptotic pathway in glioblastoma. Carcinogenesis.

[8] Nicholson B, Suresh Kumar KG (2011). The multifaceted roles of USP7: new therapeutic opportunities. Cell Biochem Biophys.

[9] Peterson LF, Sun H, Liu Y, Potu H, Kandarpa M, Ermann M, Courtney SM, Young M, Showalter HD, Sun D, Jakubowiak A, Malek SN, Talpaz M (2015). Targeting deubiquitinase activity with a novel small-molecule inhibitor as therapy for B-cell malignancies. Blood.

[10] Tian Z, D'Arcy P, Wang X, Ray A, Tai YT, Hu Y, Carrasco RD, Richardson P, Linder S, Chauhan D, Anderson KC (2014). A novel small molecule inhibitor of deubiquitylating enzyme USP14 and UCHL5 induces apoptosis in multiple myeloma and overcomes bortezomib resistance. Blood.

[11] Sippl W, Collura V, Colland F (2011). Ubiquitin-specific proteases as cancer drug targets. Future Oncol.

[12] Pal A, Young MA, Donato NJ (2014). Emerging potential of therapeutic targeting of ubiquitin-specific proteases in the treatment of cancer. Cancer Res.

[13] Li M, Brooks CL, Kon N, Gu W (2004). A dynamic role of HAUSP in the p53-Mdm2 pathway. Mol Cell.

[14] Spardy N, Covella K, Cha E, Hoskins EE, Wells SI, Duensing A, Duensing S (2009). Human papillomavirus 16 E7 oncoprotein attenuates DNA damage checkpoint control by increasing the proteolytic turnover of claspin. Cancer Res.

[15] van der Horst A, de Vries-Smits AM, Brenkman AB, van Triest MH, van den Broek N, Colland F, Maurice MM, Burgering BM (2006). FOXO4 transcriptional activity is regulated by monoubiquitination and USP7/HAUSP. Nat Cell Biol.

[16] Morotti A, Panuzzo C, Crivellaro S, Pergolizzi B, Familiari U, Berger AH, Saglio G, Pandolfi PP (2014). BCR-ABL disrupts PTEN nuclear-cytoplasmic shuttling through phosphorylation-dependent activation of HAUSP. Leukemia.

[17] Felle M, Joppien S, Nemeth A, Diermeier S, Thalhammer V, Dobner T, Kremmer E, Kappler R, Langst G (2011). The USP7/Dnmt1 complex stimulates the DNA methylation activity of Dnmt1 and regulates the stability of UHRF1. Nucleic Acids Res.

[18] Rodriguez-Rodriguez R (2014). Oleanolic Acid And Related Triterpenoids From Olives On Vascular Function: Molecular Mechanisms And Therapeutic Perspectives. Curr Med Chem.

[19] Wang YY, Yang YX, Zhe H, He ZX, Zhou SF (2014). Bardoxolone methyl (CDDO-Me) as a therapeutic agent: an update on its pharmacokinetic and pharmacodynamic properties. Drug Des Devel Ther.

[20] Wang YY, Zhe H, Zhao R (2014). Preclinical evidences toward the use of triterpenoid CDDO-Me for solid cancer prevention and treatment. Mol Cancer.

[21] Shanmugam MK, Dai X, Kumar AP, Tan BK, Sethi G, Bishayee A (2014). Oleanolic acid and its synthetic derivatives for the prevention and therapy of cancer: preclinical and clinical evidence. Cancer Lett.

[22] Duan Z, Ames RY, Ryan M, Hornicek FJ, Mankin H, Seiden MV (2009). CDDO-Me, a synthetic triterpenoid, inhibits expression of IL-6 and Stat3 phosphorylation in multi-drug resistant ovarian cancer cells. Cancer Chemother Pharmacol.

[23] Gao X, Liu Y, Deeb D, Arbab AS, Guo AM, Dulchavsky SA, Gautam SC (2011). Synthetic oleanane triterpenoid, CDDO-Me, induces apoptosis in ovarian cancer cells by inhibiting prosurvival AKT/NF-kappaB/mTOR signaling. Anticancer Res.

[24] Liby KT, Sporn MB (2012). Synthetic oleanane triterpenoids: multifunctional drugs with a broad range of applications for prevention and treatment of chronic disease. Pharmacol Rev.

[25] Morris GM, Goodsell DS, Halliday RS, Huey R, Hart WE, Belew RK, Olson AJ (1998). Automated docking using a Lamarckian genetic algorithm and an empirical binding free energy function. J Comput Chem.

[26] Molland K, Zhou Q, Mesecar AD (2014). A 2. 2 angstrom resolution structure of the USP7 catalytic domain in a new space group elaborates upon structural rearrangements resulting from ubiquitin binding. Acta Crystallogr F.

[27] Gao X, Liu Y, Deeb D, Liu P, Liu A, Arbab AS, Gautam SC (2013). ROS mediate proapoptotic and antisurvival activity of oleanane triterpenoid CDDO-Me in ovarian cancer cells. Anticancer Res.

[28] Vikhanskaya F, Erba E, D'Incalci M, Broggini M (1994). Introduction of wild-type p53 in a human ovarian cancer cell line not expressing endogenous p53. Nucleic Acids Res.

[29] Sasaki H, Sheng Y, Kotsuji F, Tsang BK (2000). Down-regulation of X-linked inhibitor of apoptosis protein induces apoptosis in chemoresistant human ovarian cancer cells. Cancer Res.

[30] Park YA, Lee JW, Kim HS, Lee YY, Kim TJ, Choi CH, Choi JJ, Jeon HK, Cho YJ, Ryu JY, Kim BG, Bae DS (2014). Tumor suppressive effects of bromodomain-containing protein 7 (BRD7) in epithelial ovarian carcinoma. Clin Cancer Res.

[31] Martinez Molina D, Jafari R, Ignatushchenko M, Seki T, Larsson EA, Dan C, Sreekumar L, Cao Y, Nordlund P (2013). Monitoring drug target engagement in cells and tissues using the cellular thermal shift assay. Science.

[32] Jafari R, Almqvist H, Axelsson H, Ignatushchenko M, Lundback T, Nordlund P, Martinez Molina D (2014). The cellular thermal shift assay for evaluating drug target interactions in cells. Nat Protoc.

[33] Lomenick B, Jung G, Wohlschlegel JA, Huang J (2011). Target identification using drug affinity responsive target stability (DARTS). Curr Protoc Chem Biol.

[34] Liby K, Royce DB, Williams CR, Risingsong R, Yore MM, Honda T, Gribble GW, Dmitrovsky E, Sporn TA, Sporn MB (2007). The synthetic triterpenoids CDDO-methyl ester and CDDO-ethyl amide prevent lung cancer induced by vinyl carbamate in A/J mice. Cancer Res.

[35] Chen ST, Okada M, Nakato R, Izumi K, Bando M, Shirahige K (2015). The Deubiquitinating Enzyme USP7 Regulates Androgen Receptor Activity by Modulating Its Binding to Chromatin. J Biol Chem.

[36] Cheng C, Niu C, Yang Y, Wang Y, Lu M (2013). Expression of HAUSP in gliomas correlates with disease progression and survival of patients. Oncol Rep.

[37] Becker K, Marchenko ND, Palacios G, Moll UM (2008). A role of HAUSP in tumor suppression in a human colon carcinoma xenograft model. Cell Cycle.

[38] Colland F (2010). The therapeutic potential of deubiquitinating enzyme inhibitors. Biochem Soc Trans.

[39] Altun M, Kramer HB, Willems LI, McDermott JL, Leach CA, Goldenberg SJ, Kumar KG, Konietzny R, Fischer R, Kogan E, Mackeen MM, McGouran J, Khoronenkova SV (2011). Activity-based chemical proteomics accelerates inhibitor development for deubiquitylating enzymes. Chem Biol.

[40] Colland F, Formstecher E, Jacq X, Reverdy C, Planquette C, Conrath S, Trouplin V, Bianchi J, Aushev VN, Camonis J, Calabrese A, Borg-Capra C, Sippl W (2009). Small-molecule inhibitor of USP7/HAUSP ubiquitin protease stabilizes and activates p53 in cells. Mol Cancer Ther.

[41] Reverdy C, Conrath S, Lopez R, Planquette C, Atmanene C, Collura V, Harpon J, Battaglia V, Vivat V, Sippl W, Colland F (2012). Discovery of specific inhibitors of human USP7/HAUSP deubiquitinating enzyme. Chem Biol.

[42] Wei W, Ma C, Cao Y, Yang L, Huang Z, Qin D, Chen Y, Liu C, Xia L, Wang T, Lei H, Yu Y, Huang M (2016). Identification of H7 as a novel peroxiredoxin I inhibitor to induce differentiation of leukemia cells. Oncotarget.

[43] Jazirehi AR, Arle D, Wenn PB (2012). UHRF1: a master regulator in prostate cancer. Epigenomics.

[44] Daskalos A, Oleksiewicz U, Filia A, Nikolaidis G, Xinarianos G, Gosney JR, Malliri A, Field JK, Liloglou T (2011). UHRF1-mediated tumor suppressor gene inactivation in nonsmall cell lung cancer. Cancer.

[45] Mudbhary R, Hoshida Y, Chernyavskaya Y, Jacob V, Villanueva A, Fiel MI, Chen X, Kojima K, Thung S, Bronson RT, Lachenmayer A, Revill K, Alsinet C (2014). UHRF1 overexpression drives DNA hypomethylation and hepatocellular carcinoma. Cancer Cell.

[46] Wu SM, Cheng WL, Liao CJ, Chi HC, Lin YH, Tseng YH, Tsai CY, Chen CY, Lin SL, Chen WJ, Yeh YH, Huang CY, Chen MH (2015). Negative modulation of the epigenetic regulator, UHRF1, by thyroid hormone receptors suppresses liver cancer cell growth. Int J Cancer.

[47] Bronner C, Achour M, Arima Y, Chataigneau T, Saya H, Schini-Kerth VB (2007). The UHRF family: oncogenes that are drugable targets for cancer therapy in the near future?. Pharmacol Ther.

[48] Alhosin M, Sharif T, Mousli M, Etienne-Selloum N, Fuhrmann G, Schini-Kerth VB, Bronner C (2011). Down-regulation of UHRF1, associated with re-expression of tumor suppressor genes, is a common feature of natural compounds exhibiting anti-cancer properties. J Exp Clin Cancer Res.

[49] Yan F, Wang X, Shao L, Ge M, Hu X (2015). Analysis of UHRF1 expression in human ovarian cancer tissues and its regulation in cancer cell growth. Tumour Biol.

