# Supplementary material for: CDDO-Me reveals USP7 as a novel target in ovarian cancer cells
Source: Oncotarget. 2016 Oct 21;7(47):77096–109. doi: 10.18632/oncotarget.12801 (PMC5363571; doi:10.18632/oncotarget.12801)
Supplement: Supplementary file 1 [file oncotarget-07-77096-s001.pdf]

CDDO-Me reveals USP7 as a novel target in ovarian cancer cells

Supplementary Materials

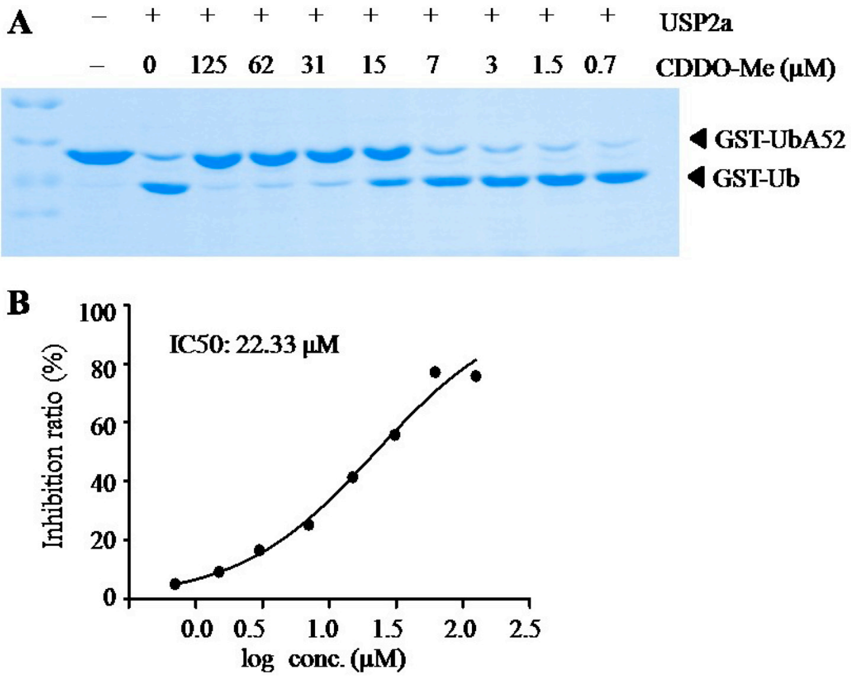

Supplementary Figure S1: CDDO-Me inhibits USP2 activity *in vitro*. In the *in vitro* gel-based USP2 activity assay, various concentrations of CDDO-Me were pre-incubated with USP2 before GST-UBA52 was added. After incubation, the reactions were stopped, and the products were separated by 12% SDS-PAGE and visualised by Coomassie brilliant blue G250 (**A**), and the IC_{50} was determined with the graphpad prism software (**B**).

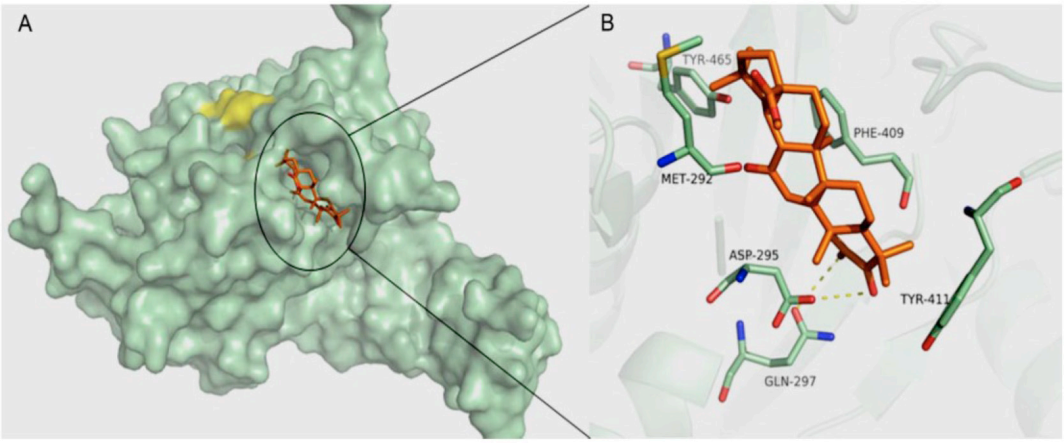

Supplementary Figure S2: Predicted conformation of CDDO-Me in the binding pocket of USP7 catalytic domain. (**A**) The molecular surface of USP7 is shown in green (catalytic triad region is colored yellow). CDDO-Me is shown in orange sticks. (**B**) Residues provided for interactions with CDDO-Me in USP7 are shown in green sticks. The dashed lines in yellow represent hydrogen bonds.

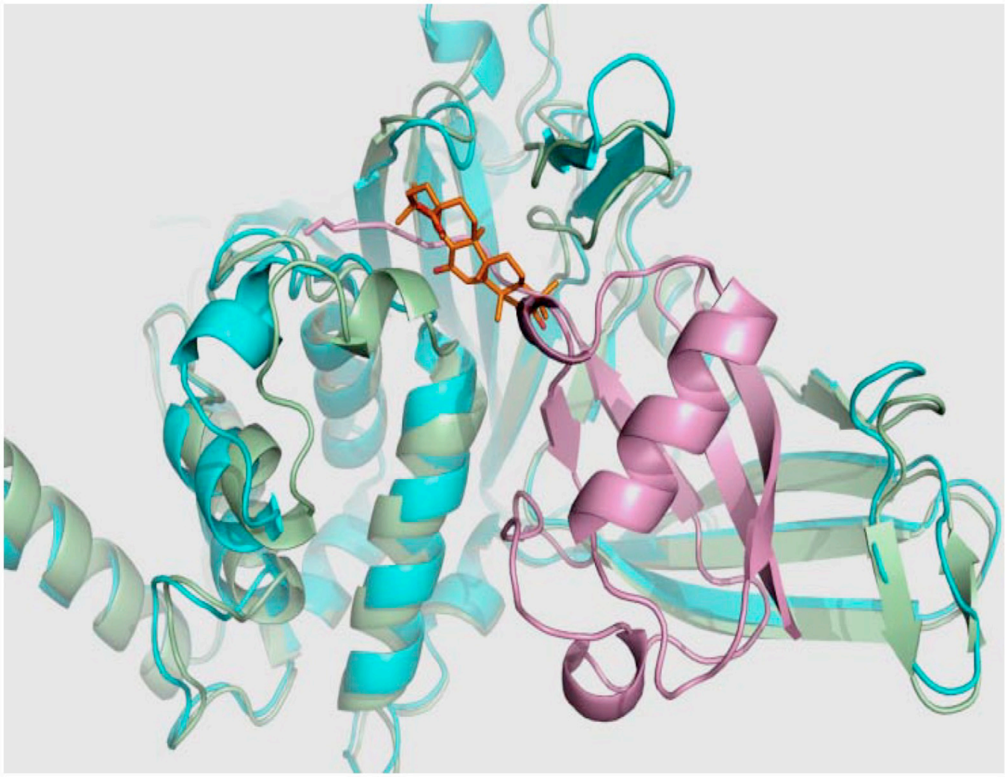

Supplementary Figure S3: Alignment of ubiquitin-bound USP7 (PDB code: 1NBF, green cartoon) and CDDO-Me-bound apo-form of USP7 (cyan cartoon). Ubiquitin is shown in pink cartoon. CDDO-Me is shown in orange sticks. Residues provided for interactions with CDDO-Me in USP7 are shown in green sticks. The dashed lines in yellow represent hydrogen bonds.

Supplementary Table S1: Screening of compounds with USP7 inhibitory activity. See Supplementary_Table_S1

Supplementary Table S2: USP7 RNAi Target sequences

	Sequence
ShUSP7#1	5′-TGCGAAATCTGCCATGGAA-3′
ShUSP7#2	5′-CTCAGAACCCTGTGATCAA-3′
ShUSP7#3	5′-CGTTCAGTCGTCGTATTAG-3′
ShUSP7#4	5′-CACGAATCAGCTACGAAAG-3′
