# Supplementary material for: CDDO-Me reveals USP7 as a novel target in ovarian cancer cells
Source: Oncotarget. 2016 Oct 21;7(47):77096–109. doi: 10.18632/oncotarget.12801 (PMC5363571; doi:10.18632/oncotarget.12801)
Supplement: Supplementary file 2 [file oncotarget-07-77096-s002.doc]

**Supplementary Materials**

**Supplementary Table S1: Screening of compounds with USP7 inhibitory activity**

| Number | Compounds Name | % Inhibition at 50 μM |
| --- | --- | --- |
| 1 | Moroxydine HCl | 17.2 |
| 2 | Clindamycin HCl | <5 |
| 3 | Felbamate | <5 |
| 4 | Cyclosporin a | <5 |
| 5 | Donepezil HCl | <5 |
| 6 | Lincomycin | <5 |
| 7 | Lomofungin | <5 |
| 8 | Mycophenolic acid | <5 |
| 9 | Novobiocin Na | <5 |
| 10 | Rapamycin | <5 |
| 11 | Spectinomycin | <5 |
| 12 | Bepridil | <5 |
| 13 | Amiodarone | <5 |
| 14 | Nicardipine | <5 |
| 15 | Pimozide | <5 |
| 16 | Loperamide | <5 |
| 17 | Fluspirilene | <5 |
| 18 | Tolbutamide | <5 |
| 19 | Pinacidil | <5 |
| 20 | Glipizide | <5 |
| 21 | Phentolamine HCl | <5 |
| 22 | Quinine | 16.9 |
| 23 | Flufenamic acid | <5 |
| 24 | Propafenone | 78.2 |
| 25 | Phenytoin | <5 |
| 26 | Procainamide | <5 |
| 27 | Lidocaine | <5 |
| 28 | Flecainide | <5 |
| 29 | Rosiglitazone maleate | <5 |
| 30 | Amantadine HCl | <5 |
| 31 | Xamoterol hemifumarate | <5 |
| 32 | Clenbuterol | <5 |
| 33 | Procaterol hcl | <5 |
| 34 | Salbutamol sulfate | <5 |
| 35 | Pindolol | <5 |
| 36 | Cimaterol | <5 |
| 37 | Dobutamine HCl | <5 |
| 38 | Pronethalol HCl | 8.3 |
| 39 | Sotalol HCl | <5 |
| 40 | Maprotiline HCl | 11.3 |
| 41 | Nisoxetine HCl | <5 |
| 42 | Canthaxanthin | <5 |
| 43 | Pilocarpine HCl | <5 |
| 44 | Ipratropium Br | <5 |
| 45 | Pirenzepine 2HCl | <5 |
| 46 | Telenzepine 2HCl | <5 |
| 47 | Tropicamide | <5 |
| 48 | Pancuronium Br | <5 |
| 49 | Ivermectin | <5 |
| 50 | Physostigmine sulfate | <5 |
| 51 | Nialamide | <5 |
| 52 | Haloperidol HCl | <5 |
| 53 | Piribedil HCl | <5 |
| 54 | Mepyramine maleate | <5 |
| 55 | Trans-triprolidine HCl | <5 |
| 56 | Cimetidine | <5 |
| 57 | Tiotidine | 11.6 |
| 58 | Zonisamide | <5 |
| 59 | Zoledronic acid | <5 |
| 60 | Naltrexone HCl | <5 |
| 61 | zolmitriptan | <5 |
| 62 | Memantine HCl | <5 |
| 63 | Aniracetam | <5 |
| 64 | Riluzole HCl | <5 |
| 65 | Propofol | <5 |
| 66 | Mianserin hcl | 21.3 |
| 67 | Aminophylline | <5 |
| 68 | Furafylline | <5 |
| 69 | nateglinide | <5 |
| 70 | Isoproterenol HCl (rac) | <5 |
| 71 | Acetylcholine Cl | <5 |
| 72 | Atropine sulfate | <5 |
| 73 | Decamethonium 2Br | <5 |
| 74 | Mecamylamine HCl | <5 |
| 75 | Neostigmine Br | <5 |
| 76 | Strychnine HCl | <5 |
| 77 | Tubocurarine Cl (+) | <5 |
| 78 | Butaclamol (+) | <5 |
| 79 | Apomorphine r (-) | <5 |
| 80 | Chlorpromazine HCl | <5 |
| 81 | Domperidone | <5 |
| 82 | Fluphenazine 2HCl | <5 |
| 83 | Pergolide mesylate | <5 |
| 84 | Raclopride l-tartrate s(-) | <5 |
| 85 | Risperidone | <5 |
| 86 | Sulpiride s (-) | <5 |
| 87 | Diphenhydramine HCl | <5 |
| 88 | Promethazine HCl | 8.9 |
| 89 | Ranitidine HCl | <5 |
| 90 | Naltrindole HCl | <5 |
| 91 | Idazoxan | <5 |
| 92 | Spiperone | <5 |
| 93 | Epinephrine-(+)-tartrate l (-) | <5 |
| 94 | Xylazine HCl | <5 |
| 95 | Norepinephrine-(+)-tartrate l (-) | <5 |
| 96 | Quetiapine fumarate | <5 |
| 97 | Alprenolol HCl | <5 |
| 98 | Imipramine HCl | 26.5 |
| 99 | Amoxapine | 16.8 |
| 100 | Mesulergine HCl | <5 |
| 101 | Amfebutamone | <5 |
| 102 | Metoclopramide HCl | <5 |
| 103 | Amorolfine | <5 |
| 104 | Clemastine fumarate | <5 |
| 105 | Vardenafil | <5 |
| 106 | Linezolid | <5 |
| 107 | Docetaxil | <5 |
| 108 | Olopatadine | <5 |
| 109 | Manidipine | <5 |
| 110 | Tolcapone | 28.6 |
| 111 | Gestrinone | <5 |
| 112 | Olmesartan | <5 |
| 113 | Aripiprazole | <5 |
| 114 | Candesartan | <5 |
| 115 | Butenafine | 24.9 |
| 116 | Dorzolamide | <5 |
| 117 | Escitalopram | <5 |
| 118 | Eprosartan | 6.2 |
| 119 | Sodium phenylacetate | <5 |
| 120 | Ozagrel | <5 |
| 121 | Uosolic acid | 75.2 |
| 122 | Corosolic acid | 43.2 |
| 123 | Asiatic acid | <5 |
| 124 | Madecassic acid | <5 |
| 125 | Oleanolic acid | 69.2 |
| 126 | Hederagenin | 36.5 |
| 127 | Bayogenin | <5 |
| 128 | Polygalacic acid | <5 |
| 129 | Pristimerin | 39.1 |
| 130 | Glycyrrhetic acid | <5 |
| 131 | Senegenin | <5 |
| 132 | Lupeol | <5 |
| 133 | Betulinic acid | 25.8 |
| 134 | Ibudilast | <5 |
| 135 | 2-cyano-3,12-dioxoolen-1,9-dien-28-oic acid (CDDO-Me) | 91.5 |
| 136 | Idarubicin | <5 |
| 137 | Montelukast | 76.3 |
| 138 | Deprenyl | <5 |
| 139 | Vatalanib | <5 |
| 140 | Fumagillin | <5 |
| 141 | Exemestane | <5 |
| 142 | Dinoprostone | 6.5 |
| 143 | Metformin | 8.6 |
| 144 | Anagrelide | 8.4 |
| 145 | Dofetilide | <5 |
| 146 | Erlotinib | <5 |
| 147 | Lobeline | <5 |
| 148 | Melatonin | <5 |
| 149 | Dinoprost | <5 |
| 150 | Verapamil | <5 |
| 151 | Niguldipine HCl | <5 |
| 152 | Flunarizine-2HCl | <5 |
| 153 | Gabapentin | <5 |
| 154 | Felodipine | 7.9 |
| 155 | Cilnidipine | <5 |
| 156 | Phenoxybenzamine HCl | <5 |
| 157 | Trifluoperazine 2HCl | <5 |
| 158 | Denbufylline | <5 |
| 159 | Miltefosine | 6.5 |
| 160 | Latanoprost | 6.9 |
| 161 | Ouabain | <5 |
| 162 | Clopamide | <5 |
| 163 | Molsidomine | <5 |
| 164 | Pravadoline | <5 |
| 165 | Captopril | <5 |
| 166 | Docebenone | <5 |
| 167 | Tranylcypromine | <5 |
| 168 | Piroxicam | <5 |
| 169 | Moxifloxacin HCl | <5 |
| 170 | Troleandomycin | <5 |
| 171 | Carbidopa | <5 |
| 172 | Nimesulide | <5 |
| 173 | Ketoprofen | <5 |
| 174 | Meloxicam | <5 |
| 175 | Terbinafine HCl | 8.6 |
| 176 | Sodium phenylbutyrate | <5 |
| 177 | Ergothioneine | <5 |
| 178 | Ambroxol | <5 |
| 179 | Idebenone | <5 |
| 180 | Mevastatin | <5 |
| 181 | Simvastatin | <5 |
| 182 | Suramin sodium | <5 |
| 183 | Goserelin acetate | <5 |
| 184 | Guaiacol | <5 |
| 185 | Retinoic acid | <5 |
| 186 | Troglitazone | 5.3 |
| 187 | Bezafibrate | <5 |
| 188 | Raloxifene HCl | <5 |
| 189 | Camptothecin | <5 |
| 190 | Plicamycin | <5 |
| 191 | Rifampicin | <5 |
| 192 | Etoposide | <5 |
| 193 | Mitomycin c | 31.5 |
| 194 | Puromycin 2HCl | <5 |
| 195 | Delavirdine mesylate | <5 |
| 196 | 10-hydroxycamptothecin | <5 |
| 197 | Daunorubicin HCl | <5 |
| 198 | Doxorubicin HCl | <5 |
| 199 | Cetirizine 2HCl | <5 |
| 200 | Lapatinib | <5 |
| 201 | Clodronate disodium | <5 |
| 202 | Clindamycin palmitate | <5 |
| 203 | Vorinostat | <5 |
| 204 | Didanosine | <5 |
| 205 | Dolasetron | <5 |
| 206 | Enalaprilat | <5 |
| 207 | Fluvastatin Na | <5 |
| 208 | Fosinopril | <5 |
| 209 | Gemcitabine HCl | 21.5 |
| 210 | Ginkgolide a | <5 |
| 211 | Granisetron | <5 |
| 212 | Ricobendazole | <5 |
| 213 | Sulbactam | <5 |
| 214 | Thiamphenicol glycinate | <5 |
| 215 | Tulobuterol | 16.5 |
| 216 | Vinorelbine | 45.3 |
| 217 | Vindesine | <5 |
| 218 | Salmeterol | <5 |
| 219 | Vincristine sulfate | <5 |
| 220 | Acemetacin | 19.8 |
| 221 | Acetylsalicylic acid | <5 |
| 222 | Acipimox | <5 |
| 223 | Aceclofenac | <5 |
| 224 | Acycloguanosine | <5 |
| 225 | 3'-azido-3'-deoxythymidine | <5 |
| 226 | Allopurinol | <5 |
| 227 | Alendronate | <5 |
| 228 | Clarithromycin | <5 |
| 229 | Climbazole | <5 |
| 230 | Clinafloxacin HCl | <5 |
| 231 | Clomiphene citrate | <5 |
| 232 | Amisulpride | <5 |
| 233 | Clopidogrel sulfate | <5 |
| 234 | Clobetasol propionate | 42.3 |
| 235 | Orphenadrine citrate | <5 |
| 236 | Corticosterone | <5 |
| 237 | Crotamiton | <5 |
| 238 | Cyclophosphamide monohydrate | <5 |
| 239 | Cyproterone acetate | 28.6 |
| 240 | Cyclocytidine HCl | 29.5 |
| 241 | Cytarabine | <5 |
| 242 | Dacarbazine | <5 |
| 243 | Danazol | <5 |
| 244 | Dehydroepiandrosterone | <5 |
| 245 | Desloratadine | <5 |
| 246 | Dextromethorphan HBr | 41.3 |
| 247 | Diclofenac, Na | <5 |
| 248 | 2',3' - dideoxycytidine | <5 |
| 249 | Diethylstilbestrol | 30.6 |
| 250 | Diflunisal | 31.5 |
| 251 | Disulfiram | <5 |
| 252 | Doxazosin mesylate | <5 |
| 253 | Doxifluridine | <5 |
| 254 | Doxofylline | <5 |
| 255 | Doxycycline HCl | <5 |
| 256 | Itraconazole | <5 |
| 257 | Ketoprofen+D153 | <5 |
| 258 | Levamisole HCl | <5 |
| 259 | Levonorgestrel | <5 |
| 260 | Levodopa | <5 |
| 261 | Levofloxacin HCl | <5 |
| 262 | Leflunomide | <5 |
| 263 | Lisinopril | 30.5 |
| 264 | Lomefloxacin HCl | 8.6 |
| 265 | Loratadine | 20.4 |
| 266 | Lorglumide | <5 |
| 267 | Losartan potassium | <5 |
| 268 | Mebendazol | <5 |
| 269 | Medroxyprogesterone 17-acetate | <5 |
| 270 | Mefenamic acid | <5 |
| 271 | Melphalan | <5 |
| 272 | Methyldopa | <5 |
| 273 | Methylprednisolone | <5 |
| 274 | Metoprolol tartrate | <5 |
| 275 | Methimazole | <5 |
| 276 | Metronidazole | <5 |
| 277 | Miconazole | <5 |
| 278 | Minocycline HCl | <5 |
| 279 | Mitoxantrone 2HCl | <5 |
| 280 | Taxol | 26.9 |
| 281 | Myclobutanil | <5 |
| 282 | Nadifloxacin | <5 |
| 283 | Nabumetone | <5 |
| 284 | Naphazoline HCl | <5 |
| 285 | Nefazodone | <5 |
| 286 | Niflumic acid | <5 |
| 287 | Pregnenolone | <5 |
| 288 | Primaquine phosphate | <5 |
| 289 | Praziquantel | <5 |
| 290 | Pyrantel pamoate | 18.6 |
| 291 | Quinacrine 2HCl dihydrate | <5 |
| 292 | Quinapril HCl | <5 |
| 293 | Racecadotril | <5 |
| 294 | Scopolamine n-butylbromide | <5 |
| 295 | Scopolamine HBr | <5 |
| 296 | Secnidazole | <5 |
| 297 | Sibutramine HCl | <5 |
| 298 | Sparfloxacin | <5 |
| 299 | Spironolactone | 36.4 |
| 300 | Stanozolol | <5 |
| 301 | Streptomycin sulfate | <5 |
| 302 | Sulfadoxine | <5 |
| 303 | Sulfadiazine | <5 |
| 304 | Sulfadimethoxine | <5 |
| 305 | Sulfasalazine | <5 |
| 306 | Vecuronium Br | <5 |
| 307 | Venlafaxine HCl | <5 |
| 308 | Ketanserin tartrate | <5 |
| 309 | Tiotropium Br | <5 |
| 310 | Mesoridazine besylate | 16.5 |
| 311 | Thioridazine HCl | <5 |
| 312 | Cilostamide | <5 |
| 313 | Etazolate | <5 |
| 314 | Amrinone | <5 |
| 315 | Irsogladine maleate | <5 |
| 316 | Milrinone | <5 |
| 317 | Rolipram | <5 |
| 318 | Siguazodan | <5 |
| 319 | Trequinsin HCl | <5 |
| 320 | Vinpocetine | 38.5 |
| 321 | Zaprinast | <5 |
| 322 | Zardaverine | <5 |
| 323 | Alprostadil | <5 |
| 324 | Misoprostol | <5 |
| 325 | Argatroban | <5 |
| 326 | Cilastatin | <5 |
| 327 | Artesunate | <5 |
| 328 | Picotamide | <5 |
| 329 | Butoconazole nitrate | <5 |
| 330 | Mifepristone | <5 |
| 331 | Megestrol acetate | <5 |
| 332 | Melengestrol acetate | <5 |
| 333 | Tamoxifen citrate | <5 |
| 334 | Amprenavir | <5 |
| 335 | Aprepitant | <5 |
| 336 | Bosentan | <5 |
| 337 | Efavirenz | 29.8 |
| 338 | Taurocholic acid, sodium salt hydrate | <5 |
| 339 | Miglustat | <5 |
| 340 | Fulvestrant | <5 |
| 341 | Methysergide | <5 |
| 342 | Esmolol | <5 |
| 343 | Pantothenic acid | <5 |
| 344 | Capecitabine | <5 |
| 345 | Phenylpropanolamine | <5 |
| 346 | Succinylcholine | <5 |
| 347 | Trifluperidol 2HCl | <5 |
| 348 | L-thyroxine [(3-[4-(4-hydroxy-3,5-diiodophenoxy)-3,5-diiodophenyl]-l-alanine] | 65.3 |
| 349 | Cyproheptadine | <5 |
| 350 | Benzydamine | <5 |
